# Hospitalizations Among Adults With CKD in Public Renal Specialty Practices: A Retrospective Study From Queensland, Australia

**DOI:** 10.1016/j.xkme.2023.100700

**Published:** 2023-07-26

**Authors:** Vishal Diwan, Wendy E. Hoy, Zaimin Wang, Jianzhen Zhang, Anne Cameron, Sree K. Venuthurupalli, Robert G. Fassett, Samuel Chan, Helen G. Healy, Ken-Soon Tan, Richard Baer, Andrew J. Mallett, Nicholas Gray, Murty Mantha, Roy Cherian, Clyson Mutatiri, Krishan Madhan, George Kan, Geoffrey Mitchell, Shahadat Hossain, Danielle Wu, Thin Han, Adrian Kark, Thomas Titus, Dwarakanatan Ranganathan, Ann Bonner, Sridevi Govindarajulu, NHMRC CKD.CRE and the CKD.QLD Collaborative

**Affiliations:** 1 NHMRC CKD.CRE and CKD.QLD, Brisbane, Queensland, Australia; 2 Faculty of Medicine, The University of Queensland, Brisbane, Queensland, Australia; 3 Brisbane, Renal Service, Ipswich Hospital, West Moreton Hospital and Health Service, Ipswich, Queensland, Australia; 4 Kidney Health Service, Metro North Hospital and Health Service, Brisbane, Queensland, Australia; 5 Department of Nephrology, Logan Hospital, Logan, Queensland, Australia; 6 Nephrology, Cairns Private Hospital, Cairns, Queensland, Australia; 7 College of Medicine & Dentistry, James Cook University, Townsville, Queensland, Australia; 8 Renal Unit, The Townsville Hospital, Townsville, Queensland, Australia; 9 Renal Medicine, Sunshine Coast University Hospital, Sunshine Coast, Queensland, Australia; 10 Nephrology Service, North Mackay, Mackay, Queensland, Australia; 11 Renal Unit, Bundaberg Hospital, Wide Bay Hospital and Health Service, Bundaberg, Queensland, Australia; 12 Renal Medicine, Hervey Bay Hospital, Hervey Bay, Queensland, Australia; 13 Hervey Bay Clinical School, University of Queensland, Hervey Bay, Queensland, Australia; 14 Bundaberg Hospital, Bundaberg Central, Queensland, Australia; 15 Mackay Base Hospital, Mackay HHS, Mackay, Queensland, Australia; 16 Rockhampton Hospital, Central Queensland HHS, Rockhampton, Queensland, Australia; 17 Rural Clinical School, University of Queensland, Rockhampton, Queensland, Australia; 18 Gold Coast University Hospital, Gold Coast, Queensland, Australia; 19 School of Medicine and Dentistry, Griffith University, Gold Coast, Queensland, Australia; 20 School of Nursing and Midwifery, Griffith University Gold Coast, Queensland, Australia; 21 St Andrew’s Hospital, Toowoomba, Queensland, Australia; 22 Tasmanian Health Service Northwest, Hobart, Tasmania, Australia; 23 School of Human Movement and Nutrition Studies, the University of Queensland, Brisbane, Queensland, Australia

**Keywords:** Chronic kidney disease, epidemiology, hospital admissions, readmissions, emergency admissions, kidney replacement therapy

## Abstract

**Rationale & Objective:**

Little is known about hospital admissions in nondialysis patients with chronic kidney disease (CKD) before death or starting kidney replacement therapy (KRT).

**Study Design:**

Retrospective observational cohort study

**Setting & Participants:**

Hospitalizations among 7,201 patients with CKD from 10 public renal clinics in Queensland (QLD), enrolled in the CKD.QLD registry starting in May 2011, were followed for 25,496.34 person-years until they started receiving KRT or died, or until June 30, 2018.

**Predictors:**

Demographic and clinical characteristics of patients with CKD.

**Outcomes:**

Hospital admissions.

**Analytical Approach:**

We evaluated the association of demographic and clinical features with hospitalizations, length of hospital stay, and cost.

**Results:**

Approximately 81.5% of the patients were admitted at least once, with 42,283 admissions, costing Australian dollars (AUD) 231 million. The average number of admissions per person-year was 1.7, and the cost was AUD 9,060, 10 times and 2 times their Australian averages, respectively. Single (1-day) admissions constituted 59.2% of all the hospital episodes, led by neoplasms (largely chemotherapy), anemia, CKD-related conditions and eye conditions (largely cataract extractions), but only 14.8% of the total costs. Approximately 41% of admissions were >1-day admissions, constituting 85.2% of the total costs, with cardiovascular conditions, respiratory conditions, CKD-related conditions, and injuries, fractures, or poisoning being the dominant causes. Readmission within 30 days of discharge constituted >42% of the admissions and 46.8% costs. Admissions not directly related to CKD constituted 90% of the admissions and costs. More than 40% of the admissions and costs were through the emergency department. Approximately 19% of the hospitalized patients and 27% of the admissions did not have kidney disease mentioned as either principal or associate causes.

**Limitations:**

Variable follow-up times because of different dates of consent.

**Conclusions:**

The hospital burden of patients with CKD is mainly driven by complex multiday admissions and readmissions involving comorbid conditions, which may not be directly related to their CKD. Strategies to prevent these complex admissions and readmissions should minimize hospital costs and outcomes.

**Plain-Language Summary:**

We analyzed primary causes, types, and costs of hospitalizations among 7,201 patients with chronic kidney disease (CKD) from renal speciality clinics across Queensland, Australia, over an average follow-up of 3.54 years. The average annual cost per person was $9,060, and was the highest in those with more advanced CKD, higher age, and with diabetes. More than 85% of costs were driven by more complex hospitalizations with longer length of stay. Cardiovascular disease was the single largest contributor for hospitalizations, length of hospital stay, and total costs. Readmission within 30 days of discharge, particularly for the same disorder, and multiday admissions should be the main targets for mitigation of hospital costs in this population.

## Graphical abstract

**Figure:**
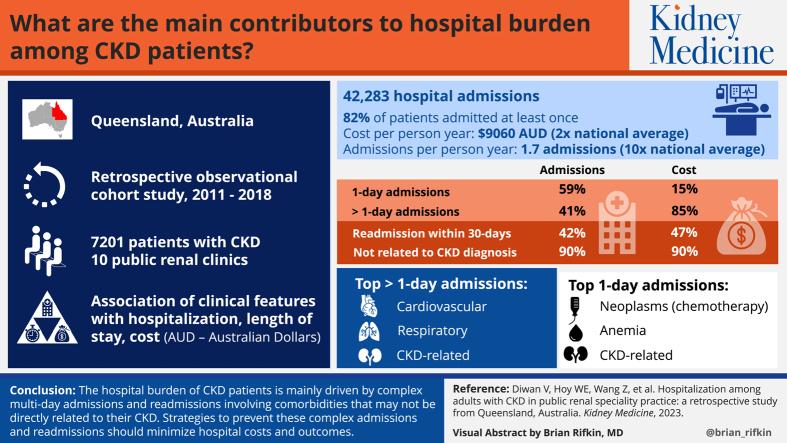

## Introduction

Chronic kidney disease (CKD) is one of the most common diseases worldwide and is associated with adverse clinical outcomes and high economic costs to health care systems.^1^ CKD either coexists with, or precipitates various additional conditions which add to the burden in the health care system as documented worldwide^2^ and in Australia,^3^^,^^4^ and the rates are continuously increasing.^5^ CKD hospitalizations, excluding kidney replacement therapy (KRT), accounted for 3.3% of all hospitalizations in Australia in 2019-2020.^6^ The cost of dialysis has been well defined; however, the cost associated with earlier, more prevalent stages of CKD is not well understood. A study among the US population reported more than 3 times higher annualized inpatient costs for admissions, excluding dialysis among patients with CKD compared with patients without CKD ($5,249 vs $1,676).^7^ Another US study reported an exponential increase in annualized inpatient Medicare costs by more advanced CKD stages, in patients with CKD not requiring dialysis (2, 3A, 3B, 4-5: $5,309, $10,088, $15,319, and $32,018, respectively) compared with patients without CKD ($2,056).^8^ Similar results have been reported from Canada^9^ and Sweden.^10^ However, the situation in Australia is still not clear.

Wyld et al^3^ used 2004-2005 AusDiab study data inflated to 2012 to estimate the cost associated with CKD in Australian patients aged 25 years or above. They reported higher direct health care costs (ambulatory services, hospitalization, and medication) in nondialysis patients with CKD compared with non-CKD patients and increasing costs with more advanced CKD stages. However, much detail about nondialysis CKD hospital admissions, including the primary diagnoses, is still not described in Australia.

It is well established that multiple risk factors, including male sex, aging, hypertension, diabetes, obesity, cardiovascular disease (CVD), and proteinuria, contribute to the faster progression of CKD, with a higher number of hospital admissions^11^ and higher hospital costs.^1^^,^^11^^,^^12^ According to the Australian Institute of Health and Welfare National Hospital Morbidity Database, there were approximately 1.8 million hospitalizations with CKD recorded as the principal or additional diagnosis in 2017-2018, representing 16% of all hospitalizations in Australia; 21% of these hospitalizations were nondialysis hospitalizations.^13^ Furthermore, the number of hospitalizations for CKD as a principal diagnosis (excluding patients receiving regular dialysis) had more than doubled between 2000-2001 and 2017-2018 from 24,100-51,300. Over the same period, the age-standardized rate increased by 54% (126 and 194 per 100,000 population, respectively).^13^ To understand and explore avenues for minimization of CKD-related hospital burden among patients not requiring dialysis, it is important to better understand the costs, causes, and patterns of hospitalizations.

A study from the United States reported that readmissions within 30 days by patients with kidney failure not requiring dialysis contributed 35%-36% of total inpatient costs, and of this cost, patients with CKD stages 3a, 3b, and 4-5 contributed 18%-33%.^8^ However, there is limited information available on causes of such readmissions and, to the best of our knowledge, no data is available from the Australian population.

We followed a group of patients across Queensland with established nondialysis CKD to ascertain the etiology, patterns, and costs of hospitalizations.

## Methods

Chronic Kidney Disease Queensland (CKD.QLD) is a programme for surveillance and research of CKD among the state-funded renal specialty services of Queensland Health. It includes the first and only registry of patients with CKD in Australia.^14^ People aged 18 years or above with an established diagnosis of CKD from 10 public renal clinics in Queensland Health facilities were recruited with informed consent and followed from the date of consent between January 1, 2011, to June 30, 2018, until the end points of death, the start of KRT or until the censor date of June 30, 2018. We have recently described the characteristics of these patients demonstrating variation in CKD by causes, age, indigenous status, the number of comorbid conditions, the prevalence of obesity, and the differences between patients who started receiving KRT and patients who died without receiving KRT.^15^

The diagnosis of CKD was based on international guidelines.^16^ Patients already receiving KRT were omitted, because all patients receiving KRT are profiled in the Australia and New Zealand Dialysis and Transplant Registry (ANZDATA).^17^ Patients with acute kidney injury were excluded unless they subsequently developed CKD and met the diagnostic criteria for CKD. The Statistical Analysis and Linkage Unit, Queensland Health, linked CKD.QLD registry data to the following collections: Queensland Hospital Admitted Patient Data Collection, Queensland Activity Based Funding Model Output Data, Death Registrations from the (QLD) Registry of Births, and Deaths and Marriages and Cause of Death Unit Record File from the Australian Coordinating Registry, informing on hospital admissions and deaths. We have recently published the data linkage methods.^18^ Queensland Hospital Admitted Patient Data Collection data included the admission date, length of hospital stays, and primary and associated reasons for the hospital admission.

Australian public hospitals translate all the diagnoses for hospitalizations to codes, using the *International Classification of Diseases* and related health problems, Tenth Revision, Australian Modification codes (ICD-10-AM codes).^19^ Generally, only a single principal diagnosis (ICD-10-AM code) is assigned to each hospital admission, whereas single or multiple diagnoses can be assigned as associated diagnoses.

For this analysis, the factors (ICD-10-AM codes) considered to have precipitated the hospital admissions were grouped into 6 categories according to potential disciplines and disorders that represented more patient centred and nephrology type of thinking (Table S1). The Alberta CKD registry group employed a similar approach in their article, which focussed on the complexity of patients treated by different specialists.^20^ Neoplasms and cancers included all the cancer-related and chemotherapy admissions represented in the neoplasm chapter. CVD admissions included all the conditions in the circulatory chapter except phlebitis, varices, varicose veins, hemorrhoids, and embolisms. The kidney and related category included all kidney disorders, CKD-related admissions, including diabetic CKD (which was sometimes included under “diabetes” in the chapter methods) as well as preparation for dialysis (which was not included under “kidney-related” in the chapter methods). We established a new category for “anemia-related admissions,” which included transfusions and iron infusions as well as anemia diagnoses. Rehabilitation and waiting for procedures or disposition also included rehabilitation and hospital stays while waiting for procedures, consultations, and disposition (transfer and discharge plans). A new category of “diabetes and related” was established, containing all diabetes codes except for those related to the kidney, but excludes the other endocrine and nutritional conditions in ICD chapter 4. Another new category of acute kidney injury was established, which had previously been buried in the ICD codes chapter 14. Finally, we used a combination of the above mentioned 6 categories and 11 selected specific ICD-10-AM chapters from chapter methods to compile 17 final “operational” categories: the “CKD.QLD grouping.”

All analyses were undertaken using Stata 16.2 (Stata Corp. Stata Statistical Software: Release 16.0: Stata Corp LP, 2016). All costs are expressed in AUD.

### Ethics Approval

The original registry ethics approval was granted in 2010 by the Queensland Health Central Human Research Ethics Committee (HREC/10/QHC/41) and the University of Queensland (No. 20110000290). In 2015, the ethics committee oversight and approval were transferred to the Royal Brisbane and Women’s Hospital Committee (HREC/15/QRBW/294). A Public Health Act approval (QCOS/029817/RD006802) was further granted in 2017 for the release of care data of the Queensland Health Statistical Analysis and Linkage Unit.

## Results

Between January 2011, and June 2018, 7,201 patients with CKD from 10 different renal services across Queensland were enrolled in CKD.QLD. They were followed until they started receiving KRT, or death, or until June 2018, for a total of 25,496.34 person-years. Table 1 shows the profile of patients at the time of consent. Almost 54% (3,884 of 7,201) were men, 60% (4,298 of 7,201) were aged 65 years or above, and 63.4% (4,563 of 7,201) had CKD stage 3B or more. Only 6.6% (475 of 7,201) patients were Indigenous. Proteinuria data were available for 6,317 patients, of whom 31% (1,960 of 6,317) had microproteinuria or albuminuria and almost 41% (2,581 of 6,317) had macroproteinuria or albuminuria. Body mass index data were available for 4,642 patients, of whom more than 52% (2,417 of 4,642) were overweight or obese. Postcodes were used to inform about the Index of Relative Socio-economic Disadvantage; data were available for 6,978 patients among whom, 55.7% (3,888) patients resided either in middle or higher socioeconomic areas. The leading primary kidney diagnosis was renovascular disease, followed by diabetic nephropathy. At consent, 76.9% (5,540 of 7,201) of patients had a diagnosis of at least 1 cardiovascular disorder, 47.5% (3,400 of 7,165) had diabetes, 17.1% (1,087 of 6,370) had cancer, and 22.5% (1,090 of 4,847) had chronic lung disease.

**Table 1:** Baseline Profile of CKD.QLD Patients at the Time of Consent.

Categories	No. of Patients (%) N=7,201
Sex	
Female	3,317 (46.1)
Male	3,884 (53.9)
Age category	
<35 y	390 (5.4)
≥35 to <45 y	419 (5.8)
≥45 to <55 y	764 (10.6)
≥55 to <65 y	1,330 (18.4)
≥65 to <75 y	2,086 (29.0)
≥75 y	2,212 (30.7)
CKD stages	
Stage 1	499 (6.9)
Stage 2	800 (11.1)
Stage 3A	1,339 (18.6)
Stage 3B	2,236 (31.1)
Stage 4	1,803 (25.0)
Stage 5	524 (7.3)
Indigenous status	
Indigenous	475 (6.6)
Nonindigenous	6,726 (93.4)
Proteinuria^a^	
Normal	1,776 (28.1)
Microproteinuria	1,960 (31.0)
Macroproteinuria	2,581 (40.9)
Primary renal diagnosis	
GRD	376 (5.2)
UNC	800 (11.1)
GN	861 (12.0)
Other	1,178 (16.4)
DN	1,811 (25.2)
RV	2,175 (30.2)
People with comorbid conditions	
CVD	5,404 (76.9)
Diabetes^b^	3,400 (47.2)
Cancer^c^	1,087 (17.1)
Chronic lung disease^d^	1,091 (22.5)
IRSD quintiles^e^	
Lowest	1,333 (19.1)
Low	1,757 (25.2)
Middle	1,465 (21)
High	1,905 (27.3)
Highest	518 (7.4)
BMI^f^	
Underweight	56 (1.2)
Normal	812 (17.5)
Overweight	1,357 (29.2)
Obese	1,840 (39.6)
Morbidly obese	577 (12.4)

Abbreviations: BMI, body mass index; CKD, chronic kidney disease; CVD, cardiovascular disease; DN, diabetic nephropathy; GN, glomerular nephropathy; GRD, genetic renal disease; IRSD, Index of Relative Socio-economic Disadvantage; RV, renovascular disease; UNC, unknown cause.

a For proteinuria at consent, data available for 6,317 patients.

b For diabetes status at the consent, data available for 7,165 patients

c For cancer status at consent, data available for 6,372 patients.

d For chronic lung disease status at consent, data available for 4,848 patients.

e For Index of Relative Socio-economic Disadvantage (IRSD), data available for 6,978 patients. IRSD is the ranks areas in Australia by socioeconomic factors.

f For BMI at consent, data available for 4,642 patients.

### Hospital Admissions, Total Length of Stay, and Total Costs

Table 2 shows data of admission, total length of stay (TLOS), and total costs by gender, age, stage of disease, and assigned primary cause of CKD. Among 7,201 patients, 5,871 (81.5%) patients (81.9% of males and 81.2% of females) were admitted one or more times, contributing to 42,283 admissions, a TLOS of 161,240 days, and total costs of $231,000,000. Males were represented among admissions (55.2%), TLOS (54.1%) and costs (55.4%) in similar proportions to their presence in the total cohort (53.9%), but, overall, their average hospital costs per person-year were marginally higher than that for females ($9,552 vs $8,516, respectively). Persons aged 75 years or above had the highest proportion of hospital admissions and the highest per person-year cost ($11,161). Patients with CKD stage 5 had the highest proportion of patients hospitalized and the highest annualized per person-year cost ($16,423). Patients with diabetic nephropathy had the highest proportion of persons admitted to the hospital and the highest per person-year cost ($12,948).

**Table 2:** Admissions, TLOS, and Costs by Different Categories.

Categories	Total Admissions n (%)	Admissions pp-y	TLOS (d) n (%)	TLOS pp-y	Total Costs (AUD) n (%)	Cost pp-y (AUD)
Overall	42,283	1.7	161,240	6.3	231 M	9,060
Sex						
Female	18,950 (44.8)	1.6	74,080 (45.9)	6.1	103 M (44.6)	8,516
Male	23,333 (55.2)	1.7	87,160 (54.1)	6.5	128 M (55.4)	9,552
Age						
<35 y	1,961 (4.6)	1.3	4,346 (2.7)	2.8	6.86 M (3)	4,451
≥35 to <45 y	1,765 (4.2)	1.1	4,948 (3.1)	3.0	8.47 M (3.7)	5,122
≥45 to <55 y	3,630 (8.6)	1.3	13,787 (8.8)	4.8	20.3 M (8.8)	7,104
≥55 to <65 y	7,630 (18)	1.6	26,592 (16.5)	5.4	42.0 M (18.2)	8,605
≥65 to <75 y	13,430 (31.8)	1.8	48,806 (30.3)	6.5	73.7 M (31.9)	9,883
≥75 y	13,867 (32.8)	2.0	62,761 (38.9)	8.8	79.3 M (34.3)	11,161
CKD stages						
Stage 1	1,724 (4.1)	0.9	4,595 (2.8)	2.4	7.18 M (3.1)	3,754
Stage 2	3,827 (9.1)	1.2	11,707 (7.3)	3.7	18.1 M (7.8)	5,693
Stage 3A	7,212 (17.1)	1.4	26,550 (16.5)	5.0	39.7 M (17.2)	7,457
Stage 3B	14,632 (34.6)	1.7	57,414 (35.6)	6.8	81.3 M (35.2)	9,678
Stage 4	12,032 (28.5)	2.1	50,832 (31.5)	8.8	69.8 M (30.2)	12,041
Stage 5	2,856 (6.8)	3.2	10,142 (6.3)	11.5	14.5 M (6.3)	16,423
Primary renal diagnosis						
GRD	1,636 (3.9)	1.1	3,987 (2.5)	2.7	6.02 M (2.6)	4,101
GN	3,996 (9.5)	1.2	12,177 (7.6)	3.5	18.0 M (7.8)	5,205
UNC	4,644 (11)	1.7	16,250 (10.1)	5.9	23.2 M (10)	8,437
Oth	7,423 (17.6)	1.8	26,573 (16.5)	6.3	34.7 M (15)	8,219
DN	11,582 (27.4)	2.0	49,414 (30.6)	8.5	75.1 M (32.5)	12,948
RV	13,002 (30.7)	1.7	52,839 (32.8)	6.8	73.6 M (31.9)	9,439

Abbreviations: AUD, Australian dollars; CKD, chronic kidney disease; DN, diabetic nephropathy; GRD, genetic renal disease; GN, glomerular nephropathy; pp-y, per person-year; RV, renovascular disease; TLOS, total length of stay; UNC, unknown cause.

### Etiology of Hospital Admissions, TLOS and Total Cost by CKD.QLD Groupings for Principal Diagnosis Associated With Hospital Admissions

**Figure 1:**
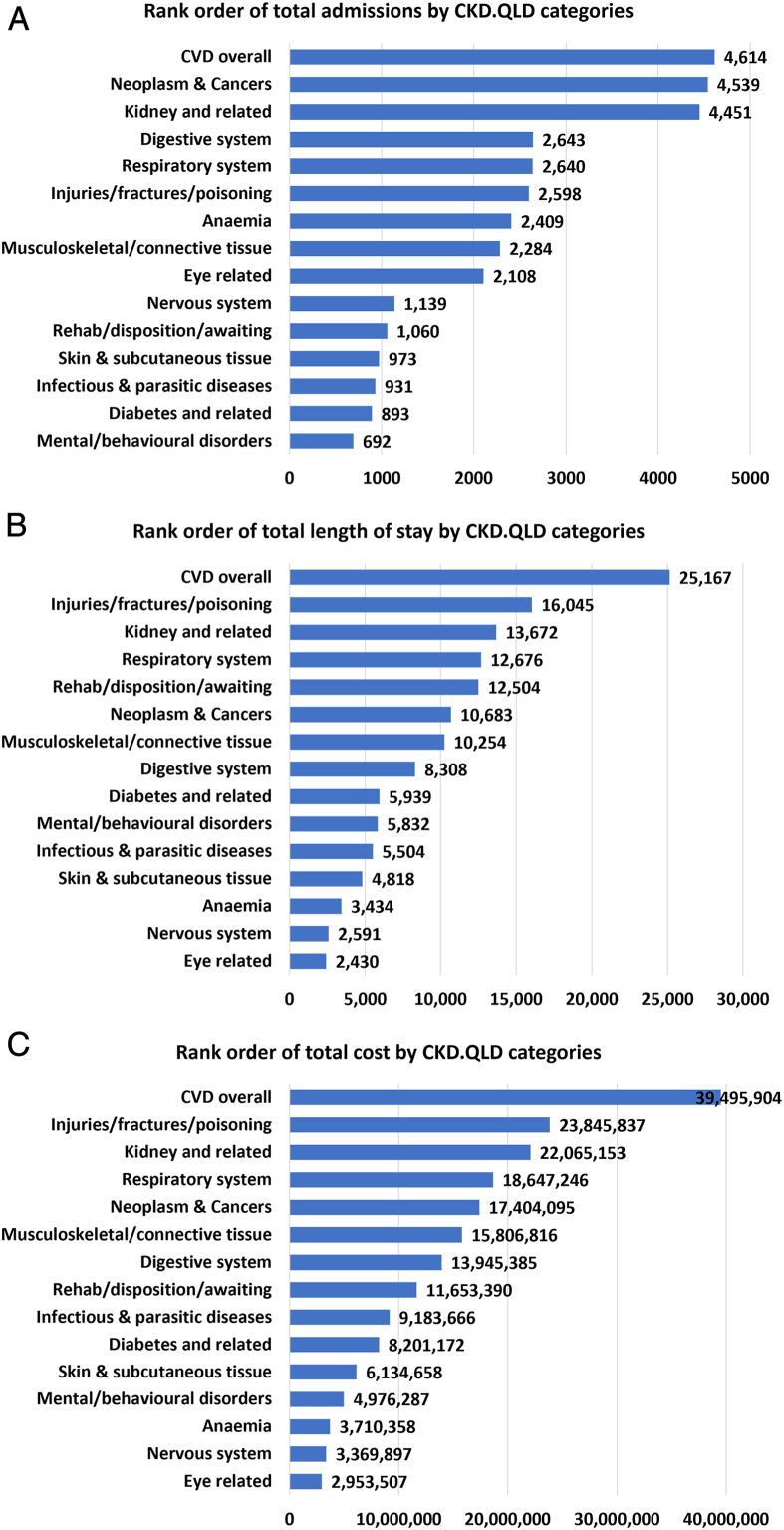
Rank orders of number of admissions, TLOS, and total costs by CKD.QLD categories. CVD, cardiovascular disease; TLOS, total length of stay.

Figure 1A shows the rank order of CKD.QLD categories by total admissions. Among 42,283 total admissions, the leading “cause” categories were CVD disorders (10.9%) as well as neoplasm and cancers (10.7%), followed by kidney-related problems (10.5%), digestive system disorders (6.3%), and respiratory systems disorders (6.2%).

Figure 1B shows the rank order of CKD.QLD categories by TLOS. Among 161,240 days of TLOS, CVD contributed the highest proportion (15.6%) followed by injuries and fractures (10%), kidney-related problems (8.5%), respiratory diseases (7.9%) and rehabilitation, disposition, or awaiting. Figure 1C shows the rank order of CKD.QLD categories by total costs of all hospitalizations. Among the total cost of $231 million, the highest proportions were for CVD overall (17.1%) followed by injuries and fractures (10.3%), kidney-related problems (9.6%), respiratory diseases (8.1%), and neoplasms and cancers (7.5%).

### Types of Admissions

Admissions were further grouped (although not necessarily mutually exclusive groups) into 1-day admissions, admissions for >1 day, readmissions within 30 days of previous discharge, and emergency admissions (admissions through the emergency department). Table 3 shows the numbers and costs of these categories of admissions. It shows that, among 42,283 admissions, costing $231 million, the majority were 1-day admissions (59.2%; 25,020 of 42,283); however, these 1-day admissions represented only 15.1% ($34.8 million of $231 million) of the total costs. Almost 41% (17,263 of 42,283) of admissions were for >1 day, but they constituted almost 85% ($196 million of $231 million) of the total costs. Among the patients who were hospitalized, 62.1% (3,645 of 5,871) had readmissions within 30 days, which constituted 42.3% (17,870 of 42,283) of the total admissions and 46.8% ($108 million of $231 million) of the total costs. Of these readmissions, 45% (8,040 of 17,870) were readmissions for the same principal diagnosis, constituting 31.6% ($34.1 million of $108 million) of their cost. Of these readmissions, 42.5% (7,601 of 17,870) were for >1-day, which constituted 89.2% ($96.3 million of $108 million) of their total costs, or 41.7% of the total costs ($231 million) of all admissions. Among the patients who had readmissions within 30 days, 2.7% (100 of 3,645) died within 30 days of their readmission representing 6.3% of the total deaths (1,583). Among all admissions, 39.7% (16,792 of 42,283) were emergency admissions, which represented 47.6% ($110 million of $231 million) of the total costs. Of these emergency admissions, almost 60% (10,016 of 16,792) were >1-day admissions, which constituted 92.7% ($96.3 million of $110 million) of the emergency admission costs, or 44.2% of the total costs ($231 million) of all the admissions.

**Table 3:** Cross Tabulation of Total Admissions and Costs by Types of Admissions.

	Total Admissions N	1-Day Admissions n (%)	>1-Day Admissions n (%)
Total			
Admissions	42,283	25,020 (59.2)	17,263 (40.8)
Cost (millions)	231	34.9 (15.2)	196 (84.8)
1-day admissions			
Admissions	25,020	25,020 (100)	0
Cost (millions)	34.9	34.9 (100)	0
>1-day admissions			
Admissions	17,263	0	17,263 (100)
Cost (millions)	196	0	196 (100)
Readmissions			
Admissions	17,870	10,269 (57.4)	7,601 (42.6)
Cost (millions)	108.3	12.1 (11.4)	96.2 (88.6)
Readmissions for same cause			
Admissions	8,040	5,888 (73.2)	2,152 (26.8)
Cost (millions)	34.1	5.78 (17.7)	28.3 (82.3)
Emergency admissions			
Admissions	16,792	6,776 (40.4)	10,016 (59.6)
Cost (millions)	110.1	8.1 (7.4)	102 (92.6)
Nonemergency admissions			
Admissions	25,491	18,244 (71.6)	7,247 (28.4)
Cost (millions)	120.9	27.1 (22.4)	93.8 (77.6)

**Figure 2:**
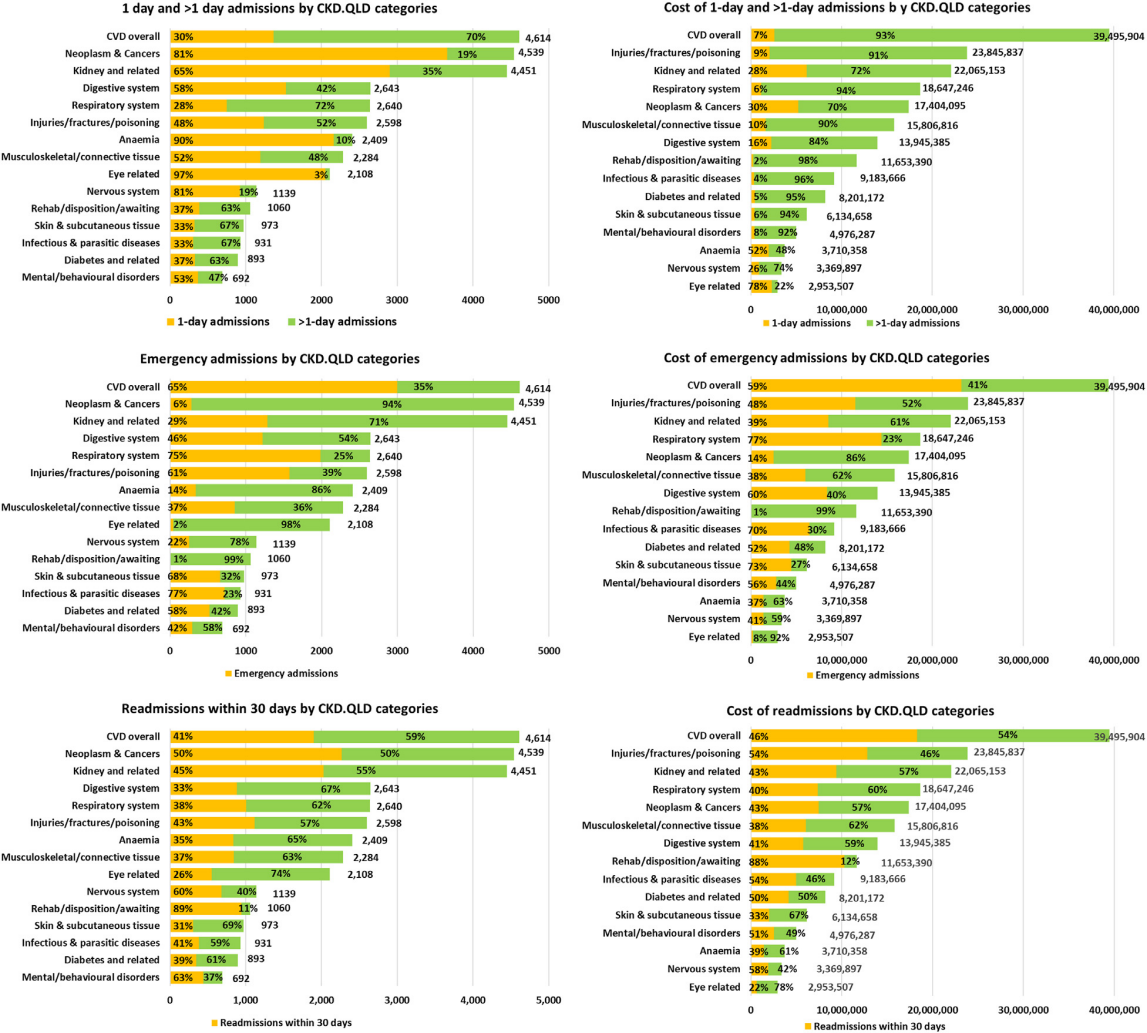
Types and costs of admissions: 1-day, >1-day, emergency, and readmissions. CVD, cardiovascular disease.

Figure 2 shows the representation of those categories of admission by CKD.QLD “cause” categories. Categories with the highest proportions of 1-day admissions were eye-related conditions (including cataract and retinal procedures), 97.3% (2,052 of 2,108); followed by anemia-related conditions, (including transfusions, infusions, and diagnostic procedures), 89.7% (2,161 of 2,409); nervous system–related conditions (mainly myasthenia gravis and inflammatory polyneuropathies), 81.2% (925 of 1,139); neoplasms and cancers (including chemotherapy, biopsies, and other diagnostic and staging procedures), 80.6% (3,660 of 4,539); and kidney and related conditions (including preparation for dialysis, CKD stage 5, and acute kidney injury), 65.2% (2,901 of 4,451). The proportion of costs expended on 1-day admissions was highest for the eye-related admissions at 77.7% (∼ $2.3 million of $2.953 million), followed by anemia, 52.4% ($1.94 million of $3.71 million); neoplasm and cancers, 29.9% ($5.2 million of $17.4 million); kidney and related conditions 27.9% ($6.17 million of $22.07 million); and nervous system–related, 26.2% ($0.9 million of $3.37 million).

Figure 2 also shows that respiratory system disorders and CVD disorders had the highest proportions of admissions of >1 day, 71.5% (1,888 of 2,640) and 70.4% (3,246 of 4,614) respectively, followed by infections and parasitic diseases, >60%, skin-related conditions (mainly cellulitis of various limbs), diabetes-related problems, and rehabilitation, disposition, or consultations. For all these categories, these longer admissions accounted for >90% of the costs.

Figure 2 also shows that the majority of admissions for infectious disorders, respiratory disorders, skin-related problems, CVD and injuries, fractures, or, poisoning were through the emergency department, and, with the questionable exception of injuries, fractures, orpoisonings, these emergency admissions accounted for most of hospital costs in those categories. Notably, emergency admissions were not common among admissions for rehabilitation, disposition, or awaiting consultations, eye-related conditions, neoplasms and cancers, as well as anemia, attesting to the forward planning and more elective nature of these admissions.

Table 4 shows CKD-related admissions and admissions for principal diagnoses related to other disorders. The “CKD-related” category represented 10.5% (4,451 of 42,283) of the total admissions, 8.5% (13,672 of 161,240) of hospital days, and 9.7% ($22.1 million of $231 million) of the total costs. Of CKD-related admissions, 65.2% (2,901 of 4,451) were 1-day admissions. It is noteworthy that admissions not directly related to CKD constituted 89.5% (37,832 of 42,283) of the total admissions, 91.5% (147,568 of 161,240) of hospital days, and 90.5% ($209 million of $231 million) of the total costs. Almost 58.5% (22,119 of 37,832) of these were 1-day admissions, but most hospital days and costs were driven by more prolonged admissions, with cardiovascular and respiratory diseases as the leading causes. After annualizing CKD-related versus non-CKD hospital admissions, the respective numbers of admissions were 0.17 versus 1.5, numbers of hospital days were 0.54 versus 5.8, and costs were $868 versus $8,196.

**Table 4:** Comparison of Direct (kidney-related) and Indirect (nonkidney-related) Admissions, TLOS, and Costs.

Variables	Kidney-Related Admissions	Non–Kidney-Related Admissions
Total admissions, (%)	4,451 (10.5%)	37,832 (89.5%)
TLOS, (%)	13,672 (8.5%)	147,568 (91.5%)
Total cost, ( %)	22,100,000 (9.7%)	209,000,000 (90.3%)
Admissions per person-year	0.17	1.5
LOS per person-year	0.54	5.8
Cost per person-year	868	8,196
1-day admissions	2,901	22,119
TLOS for 1-day admissions	2,901	22,119
Cost for 1-day admissions	6,165,000	29,000,000
>1-day admissions	1,550	15,713
TLOS for >1-day admissions	10,771	125,449
Cost for >1-day admissions	15,900,000	180,000,000

Abbreviation: TLOS, total length of stay.

Table 5 shows the admissions with kidney disease mentioned as either the principal or associated cause. Among 5,871 hospitalized patients, only 81.9% (4,808) had kidney disease-related ICD-10-AM codes mentioned. Among 42,283 total admissions, only 59.2% (25,034) of the admissions had kidney disease-related ICD-10-AM codes mentioned, although, we emphasize that everyone in this cohort had well established CKD. The recording of a CKD-related diagnosis increased with increasing severity of CKD by stage at enrolment, and was higher for emergency admissions than for other types of admissions; nonetheless, a CKD diagnosis was absent in almost 22% of the admissions of persons with CKD stage 5 disease overall, and in 16% of CKD stage 5 emergency admissions.

**Table 5:** Proportion of Admissions with Kidney Disease-Related Mentions (primary and associated).

CKD Stages	Total Admissions	All Admissions With KD Mentions		Total ED Admissions	ED Admissions With KD Mentions	
		No.	%		No.	%
1	1,724	413	24.0	619	205	33.1
2	3,827	1,286	33.6	1,369	684	50.0
3A	7,212	3,618	50.2	2,855	1,943	68.1
3B	14,632	8,784	60.0	6,025	4,558	75.7
4	12,032	8,683	72.2	5,075	4,227	83.3
5	2,856	2,250	78.8	849	715	84.2
Total	42,283	25,034	59.2	16,792	12,332	73.4

Abbreviations: CKD, chronic kidney disease; ED, emergency department; KD, kidney disease.

## Discussion

The Australian health care system includes both public and private hospitals. Public hospitals are mainly owned and managed by the state and territory governments and mainly provide acute care for short periods, although some provide long-term care, for chronic illnesses and rehabilitation. There are public psychiatric hospitals, which specialize in the care of people with mental health problems, sometimes for long periods. Private hospitals are mainly owned and managed by private organizations—either for-profit companies, or not-for-profit nongovernment organizations. They include day hospitals that provide services on a day-only basis, and hospitals that provide overnight care.^21^ All our patients were enrolled in our registry while they were receiving outpatient renal speciality care in the public health care system. We did not capture people and cannot describe patients with CKD whose outpatient care was exclusively in private renal specialty practices. However, as CKD advances, progressively higher proportion of patients with CKD seeking care previously from the private system progress into the public system, because of cost considerations, and often sparsity of private services in more remote settings. However, the QLD linked data capture includes all admissions at any hospital in Queensland (public and private), so that, once enrolled in our registry, all the hospital episodes of an individual patient are recorded, unless they move out of the state.

CKD.QLD is the first to report the hospital admissions, length of hospital stay, and the total cost among patients with CKD in Australia. In 2017-2018, Australia had approximately 5.2 million hospital admissions, excluding dialysis,^22^ which is approximately 0.2 per person-year. However, in this cohort with established CKD severe and/or complex enough to warrant renal specialty consultation, there were 1.7 admissions per person-year, which is 8 times higher than the normal average in Australia.^22^

The average per person-year cost for hospital services in Australia in 2018-2019 was $4,891 (63% of the total health expenditure).^23^ In this cohort of patients with CKD, the average per person-year cost was $9,060, which is almost double the national average, which strongly supports reports of high health care costs in patients with CKD.^3^ Our observations support reports from other countries that hospital admissions and costs are higher with advancing age,^24^^,^^25^ more advanced CKD stages^8^^,^^9^^,^^26^ and with diabetic nephropathy.^26^^,^^27^

To our knowledge, this is the first report that the preponderance of admissions among patients with CKD are 1-day admissions. Approximately 60% of the admissions in our CKD.QLD cohort were 1-day admissions, which is higher than the national average of 40% (excluding dialysis) in Australia^21^; however, these 1-day admissions constituted only 15.2% of the total costs. The 5 major causes of 1-day admissions were neoplasms and cancers (mostly chemotherapy), 14.6%; kidney-related problems (mostly preparation for dialysis), 11.6%; anemia and related disorders (mostly transfusions and infusions), 8.6%; eye conditions (mostly cataract extractions), 8.2%; and digestive disorders (gastritis, ulcers, gastrointestinal bleeding, constipation, and reflux disorders), 6.1%. CKD and cancer share strong associations^28^ with advancing age, largely explaining the association of admissions with chemotherapy, which is usually (in approximately 81% of the cases) administered in 1-day admissions. Additionally, many chemotherapeutic agents are nephrotoxic. There were also substantial numbers of admissions related to kidney damage associated with multiple myeloma. The well-known associations of CKD with anemia and with gastrointestinal bleeding are reflected in the hospital admissions for anemia, almost 90% of which were 1-day admissions. Aging and diabetes provide common pathways for the mutual connections of CKD with eye disorders, largely cataracts, and retinal conditions, and 97.3% of all hospital disorders for eye conditions were 1-day admissions. Approximately 10% of the patients in this cohort started receiving KRT, and these patients had dialysis preparation^29^ admissions, and 89% of such admissions in our cohort were 1-day admissions. The whole spectrum of gastrointestinal disorders was represented in the digestive system with no predominant conditions, but they did include conditions, such as hemorrhages, constipation, ulcers, and colitis.

The high cost of hospital admissions in this CKD cohort was largely from more complex hospital admissions for >1 day. These constituted only 41% of all admissions but approximately 85% of the total cost. They reflect frequent and serious multisystem comorbid conditions in many patients with CKD. Our data also confirm that these issues become more pronounced with more advanced CKD stage as reported by studies from other countries with higher numbers of admissions,^30^ length of hospital stay, and costs.^31^ Of these complex hospital admissions, the highest impact on the number of hospital admissions and hospital costs was from CVD, compatible with other reports.^32^^,^^33^

More than 42% of the total admissions were readmissions within 30 days of discharge, which constituted almost 47% ($108.3 million of $231 million) of the total costs. More than 42% of these readmissions were complex involving >1 day of hospital stay constituting 18% of the total admissions. Readmissions within 30 days of discharge, particularly for the same disorder, include those that are part of a scheduled management regimen, such as chemotherapy treatments, and those that were not scheduled or necessarily anticipated. The costs of the 1-day readmissions were relatively trivial, but the costs of readmissions with >1-day length of hospital stay were a major expense. To our knowledge, this is the first report showing the impact of such admissions. As found in other studies,^6^ CVD was the single largest contributor among >1-day readmissions for cost, costing $17.4 million. Planned treatment regimens involving 1-day admissions clearly represent practical and efficient service delivery. However, identifying and minimizing risks for readmissions, especially those for the same condition, is a promising area to explore for potential reduction in the burden of hospital service delivery.

Other studies from Australia^32^ and other countries^7^^,^^30^ confirm that CVD had the highest impact on hospital admissions and hospital costs among patients with CKD. Studies from other countries also confirm the significant hospital burden and costs posed by fractures,^34^ cancer chemotherapy,^35^ and dialysis preparation^29^ across the CKD spectrum.

Almost half the costs of hospital services for these patients with CKD were associated with admissions through the emergency department. This is, perhaps, a surprising statistic for a group of patients who were already in (and recruited from) a (renal) specialty health care stream. Of these emergency department admissions, the majority, 59.6%, were for >1 day, and they constituted 44.2% of the total costs. Minimization of emergency admissions is an additional avenue for further investigation and cost minimization.

Kidney disease is associated with other complex disorders, and the health service burden is conflated by these conditions.^6^ For example, in 2019-2020, in Australia, there were approximately 373,000 CKD-related nondialysis hospitalizations, of which only 14.4% had CKD mentioned as the principal diagnosis for hospital admissions.^6^ Our study confirms that the diseases coexisting with nondialysis CKD pose a greater hospital service and cost burden than CKD itself.

All the patients in the CKD.QLD registry were recruited from renal specialty practices and had established CKD at the more severe end of the CKD stage spectrum, so that an ICD-10-AM code related to kidney disease should be mentioned in all their hospital admissions.^36^ This was not the case, as has been confirmed by other hospital administrative data.^19^ Although such ascertainment improved with more severe stages of CKD, it was still lacking in overall 19% of the cases. This can compound risk for, and severity of, complications in individual patients, and impair planning and policy development for CKD more broadly. It also highlights possible under representation of kidney disease when diagnosis codes are used for identification in extensive nationwide audits.^37^

We cannot report the level of the complexity of the hospital admissions, which includes speciality visits to the patients. Also, educational details of the patients and smoking status was not available for the patients in this cohort.

The hospital burden is very high, reflecting the significant burden of morbidities in these patients with CKD. One avenue for hospitalization minimization is preventing readmissions for the same diagnosis following complex (>1 day) hospitalizations. Also, well-documented ICD codes can undoubtedly clarify the picture in a broader way for the policymakers and clinicians involved inpatient care.

## References

[1] Hill N.R., Fatoba S.T., Oke J.L. (2016). Global prevalence of chronic kidneydisease–a systematic review and meta-analysis. PloS One.

[2] Couser W.G., Remuzzi G., Mendis S., Tonelli M. (2011). The contribution of chronic kidney disease to the global burden of major noncommunicable diseases. Kidney Int.

[3] Wyld M.L., Lee C.M., Zhuo X. (2015). Cost to government and society of chronic kidney disease stage 1-5: a national cohort study. Intern Med J.

[4] Tucker P.S., Kingsley M.I., Morton R.H., Scanlan A.T., Dalbo V.J. (2014). The increasing financial impact of chronic kidney disease in australia. Int J Nephrol.

[5] (2020). Chronic Kidney Disease.

[6] Australian Institute of Health and Welfare (2022).

[7] Nichols G.A., Ustyugova A., Deruaz-Luyet A., O'Keeffe-Rosetti M., Brodovicz K.G. (2020). Health care costs by type of expenditure across eGFR stages among patients with and without diabetes, cardiovascular disease, and heart failure. J Am Soc Nephrol.

[8] Golestaneh L., Alvarez P.J., Reaven N.L. (2017). All-cause costs increase exponentially with increased chronic kidney disease stage. Am J Manag Care.

[9] Manns B., Hemmelgarn B., Tonelli M. (2019). The cost of care for people with chronic kidney disease. Can J Kidney Health Dis.

[10] Eriksson J.K., Neovius M., Jacobson S.H., Elinder C.G., Hylander B. (2016). Healthcare costs in chronic kidney disease and renal replacement therapy: a population-based cohort study in Sweden. BMJ Open.

[11] Tsai W.C., Wu H.Y., Peng Y.S. (2016). Risk factors for development and progression of chronic kidney disease: a systematic review and exploratory meta-analysis. Medicine.

[12] Saran R., Robinson B., Abbott K.C. (2018). US renal data system 2017 annual data report: epidemiology of kidney disease in the United States. Am J Kid Dis.

[13] Australian Institute of Health and Welfare (2020), Chronic kidney disease 2020.

[14] Venuthurupalli S.K., Hoy W.E., Healy H.G., Cameron A., Fassett R.G. (2017). CKD.QLD: establishment of a chronic kidney disease [CKD] registry in Queensland, Australia. BMC Nephrol.

[15] Hoy W.E., Zhang W., Zhang J. (2022). Chronic kidney disease in public renal practices in Queensland, Australia, 2011-2018. Nephrology (Carlton).

[16] National Kidney Foundation (2002). K/DOQI clinical practice guidelines for chronic kidney disease: evaluation, classification, and stratification. Am J Kidney Dis.

[17] ANZDATA Registry. 43rd Report, Chapter 1: Incidence of Renal Replacement Therapy for End Stage Kidney Disease. Australia and New Zealand Dialysis and Transplant Registry, Adelaide, Australia.2020.

[18] Zhang J., Wang Z., Cameron A. (2021). The CKD.QLD data linkage framework: chronic kidney disease and health services utilisation in Queensland, Australia. F1000Research.

[19] Ko S., Venkatesan S., Nand K., Levidiotis V., Nelson C., Janus E. (2018). International statistical classification of diseases and related health problems coding underestimates the incidence and prevalence of acute kidney injury and chronic kidney disease in general medical patients. Intern Med J.

[20] Tonelli M., Wiebe N., Manns B.J. (2018). Comparison of the complexity of patients seen by different medical subspecialists in a universal health care system. JAMA Netw Open.

[21] Australian Institute of Health and Welfare (2019).

[22] Nishikawa K., Takahashi K., Yamada R., Kinaga T., Masato M., Yamamoto M. (2017). Influence of chronic kidney disease on hospitalization, chronic dialysis, and mortality in Japanese men: a longitudinal analysis. Clin Exp Nephrol.

[23] Australian Institute of Health and Welfare (2021).

[24] Clementi A., Coppolino G., Provenzano M., Granata A., Battaglia G.G. (2021). Holistic vision of the patient with chronic kidney disease in a universalistic healthcare system. Ther Apher Dial.

[25] van Oosten M.J.M., Logtenberg S.J.J., Leegte M.J.H. (2020). Age-related difference in health care use and costs of patients with chronic kidney disease and matched controls: analysis of Dutch health care claims data. Nephrol Dial Transplant.

[26] Kent S., Schlackow I., Lozano-Kuhne J. (2015). What is the impact of chronic kidney disease stage and cardiovascular disease on the annual cost of hospital care in moderate-to-severe kidney disease?. BMC Nephrol.

[27] Nichols G.A., Vupputuri S., Lau H. (2011). Medical care costs associated with progression of diabetic nephropathy. Diabetes Care.

[28] Stengel B. (2010). Chronic kidney disease and cancer: a troubling connection. J Nephrol.

[29] London R., Solis A., Goldberg G.A., Wade S., Ryu S. (2002). Health care resource utilization and the impact of anemia management in patients with chronic kidney disease. Am J Kidney Dis.

[30] Wan E.Y.F., Chin W.Y., Yu E.Y.T. (2020). The impact of cardiovascular disease and chronic kidney disease on life expectancy and direct medical cost in a 10-year diabetes cohort study. Diabetes Care.

[31] Schrauben S.J., Chen H.Y., Lin E. (2020). Hospitalizations among adults with chronic kidney disease in the United States: a cohort study. PLoS Med.

[32] Ariyaratne T.V., Ademi Z., Duffy S.J. (2013). Cardiovascular readmissions and excess costs following percutaneous coronary intervention in patients with chronic kidney disease: data from a large multi-centre Australian registry. Int J Cardiol.

[33] Imtiaz∗ S., Qureshi R., Hamid A., Salman B., Drohlia M.F., Ahmad A. (2019). Causes of hospital admission of chronic kidney disease patient in a tertiary kidney care hospital. J Clin Nephrol.

[34] Naylor K.L., McArthur E., Leslie W.D. (2014). The three-year incidence of fracture in chronic kidney disease. Kidney Int.

[35] de Francisco A.L.M., Macia M., Alonso F. (2019). Onco-nephrology: cancer, chemotherapy and kidney. Nefrol (Engl Ed).

[36] Yong T.Y., Fok J.S., Ng P.Z. (2013). The significance of reduced kidney function among hospitalized acute general medical patients. QJM.

[37] Abraham K.A., Thompson E.B., Bodger K., Pearson M. (2012). Inequalities in outcomes of acute kidney injury in England. QJM.

