# Supplementary material for: Hospitalizations Among Adults With CKD in Public Renal Specialty Practices: A Retrospective Study From Queensland, Australia
Source: Kidney Med. 2023 Jul 26;5(9):100700. doi: 10.1016/j.xkme.2023.100700 (PMC10462882; doi:10.1016/j.xkme.2023.100700)
Supplement: Supplementary File (PDF) — Table S1 [file mmc1.pdf]

## Table S1: ICD-10AM codes for CKD.QLD categories

| | |
|---|---|
| Neoplasms and cancers | C00 to D48.9, Z03.1, Z08 to Z09, Z12 to Z12.9, Z40.0 to Z40.08, Z51.1, Z80 to Z80.9, Z85 to Z85.9, Z86.0 |
| Kidney related | E87.5 to E87.8, E09.2 to E09.29, E10.2 to E10.29, E11.2 to E11.29, E12.2, E12.20, E12.21, E13.2 to E13.29, E14.2 to E14.29, E87.5, E87.7, E87.8, I12, I12.0, I12.9, I13*, I130*, I131*, I132*, I139*, I150*, I151, K76.7, M10.3 to M10.39, N00 to N29.8, O10.2, O10.3, O26.81, R80, R82.1, R82.3, R93.4, U87.1, Z49 to Z49.2<br>*These codes are also added to CVD overall because of common pathophysiology |
| Rehab/disposition/awaiting | Z50, Z50.0, Z50.1, Z50.4, Z50.5, Z50.7, Z50.8, Z50.9, Z51, Z51.4, Z51.5, Z75.0 to Z75.49 |
| Diabetes and related | E09 to E09.1, E09.3 to E10.16, E10.3 to E11.16, E11.3 to E12.11, E12.3 to E13.16, E13.3 to E14.16, E14.3 to E14.91 |
| Anaemia | D46.0 to D46.5, D50 to D55.9, D57.0, D57.1, D58, D58.8, D58.9, D59.0 to D59.2, D59.8, D59.9, D60 to D64.9, E61.1, O99.0 to O99.04, P61.2 to P61.4 |
| CVD overall | E10.5 to E10.59, E11.5 to E11.59, E13.5 to E13.59, E14.5 to E14.59, F45.31, G45, G45.9, I11 to I11.9, I13 to I13.9, I15.1 to I15.9, I20 to I25.9, I21 to I23.8, I24.1, I25.2, I25.6, I27 to I69.8, I97.82, R00, R00.8, R93.1, T86.2, T86.3, U82, U82.1, U82.2, Y83.03, Y83.05, Z03.4, Z95.2 to Z95.9 |
